# Examining the interaction of fast-food outlet exposure and income on diet and obesity: evidence from 51,361 UK Biobank participants

**DOI:** 10.1186/s12966-018-0699-8

**Published:** 2018-07-24

**Authors:** Thomas Burgoine, Chinmoy Sarkar, Chris J. Webster, Pablo Monsivais

**Affiliations:** 1 UKCRC Centre for Diet and Activity Research (CEDAR), MRC Epidemiology Unit, University of Cambridge School of Clinical Medicine, Box 285 Institute of Metabolic Science, Cambridge Biomedical Campus, Cambridge, CB2 0QQ UK; 2 Healthy High Density Cities Lab, HKUrbanLab, University of Hong Kong, Knowles Building, Pokfulam Road, Hong Kong, Special Administrative Region China; 3 Present Address: Department of Nutrition and Exercise Physiology, Elson S. Floyd College of Medicine, Washington State University, Spokane, Washington USA

**Keywords:** Fast-food outlet exposure, Household income, Adiposity, Processed meat consumption, Interaction, UK biobank

## Abstract

**Background:**

Household income (as a marker of socioeconomic position) and neighbourhood fast-food outlet exposure may be related to diet and body weight, which are key risk factors for non-communicable diseases. However, the research evidence is equivocal. Moreover, understanding the double burden of these factors is a matter of public health importance. The purpose of this study was to test associations of neighbourhood fast-food outlet exposure and household income, in relation to frequency of consumption of processed meat and multiple measures of adiposity, and to examine possible interactions.

**Methods:**

We employed an observational, cross-sectional study design. In a cohort of 51,361 adults aged 38–72 years in Greater London, UK, we jointly classified participants based on household income (£/year, four groups) and GIS-derived neighbourhood fast-food outlet proportion (counts of fast-food outlets as a percentage of all food outlets, quartiles). Multivariable regression models estimated main effects and interactions (additive and multiplicative) of household income and fast-food outlet proportion on odds of self-reported frequent processed meat consumption (> 1/week), measured BMI (kg/m^2^), body fat (%), and odds of obesity (BMI ≥ 30).

**Results:**

Income and fast-food proportion were independently, systematically associated with BMI, body fat, obesity and frequent processed meat consumption. Odds of obesity were greater for lowest income participants compared to highest (OR = 1.54, 95% CI: 1.41, 1.69) and for those most-exposed to fast-food outlets compared to least-exposed (OR = 1.51, 95% CI: 1.40, 1.64). In jointly classified models, lowest income and highest fast-food outlet proportion in combination were associated with greater odds of obesity (OR = 2.43, 95% CI: 2.09, 2.84), with relative excess risk due to interaction (RERI = 0.03). Results were similar for frequent processed meat consumption models. There was no evidence of interaction on a multiplicative scale between fast-food outlet proportion and household income on each of BMI (*P* = 0.230), obesity (*P* = 0.054) and frequent processed meat consumption (*P* = 0.725).

**Conclusions:**

Our study demonstrated independent associations of neighbourhood fast-food outlet exposure and household income, in relation to diet and multiple objective measures of adiposity, in a large sample of UK adults. Moreover, we provide evidence of the double burden of low income and an unhealthy neighbourhood food environment, furthering our understanding of how these factors contribute jointly to social inequalities in health.

**Electronic supplementary material:**

The online version of this article (10.1186/s12966-018-0699-8) contains supplementary material, which is available to authorized users.

## Background

Unhealthy diet and high body weight are key modifiable risk factors for non-communicable diseases (NCDs) such as cardiovascular disease, type-2 diabetes and some cancers. According to the WHO’s Global Burden of Disease study, dietary risks and high body weight are two of the top three contributors to the number of years suffered with disability and morbidity in the UK [1]. Food-related ill health in the UK, driven by unhealthy diet, contributes to 10% of the combined mortality and morbidity burden [2]. Processed meat consumption, in particular, is associated with higher incidence of type-2 diabetes, coronary heart disease [3–5], and certain cancers [6].

Social inequalities in diet and obesity are established across markers of socioeconomic position (SEP) such as education, income (including *household* income [7]) and occupation [8], and may be contributing to recognised inequalities in NCD risk [9, 10]. Explanations include having less knowledge about healthy eating and fewer cooking skills [11, 12], and less time for cooking at home among socially disadvantaged groups [13]. Low-income groups, in particular, are also more price-sensitive [14], spending less on food overall and per calorie than other social groups [15].

In addition to individual-level socioeconomic determinants, neighbourhood food environments are also potentially important influences on diet and weight [16, 17], although the research evidence remains equivocal [18, 19]. Neighbourhoods where the mix of food retailing is biased towards a high proportion of fast-food outlets may be especially influential [20–22] – where fast-food might be perceived as the easier choice and therefore used more, and where greater fast-food access is not simply a function of greater access to all types of food outlet [23]. As a result, recent studies have increasingly situated fast-food access within the wider context of overall neighbourhood food retailing through adopting relative as opposed to absolute measures of exposure [24–30]. Easier access to fast-food outlets may facilitate the consumption of energy dense, nutrient poor fast foods [31, 32], which have been linked prospectively to excess weight gain [33], and associated with other cardio-metabolic risk factors [34]. Despite some fast-food outlets selling ‘healthier’ foods than others, at a population level, visits to and use of fast-food outlets has been linked to weight gain over time [35], and consumption of a less healthy diet and greater odds of obesity [36], respectively.

Fast-food access may also contribute to established social inequalities in fast-food consumption and weight [37]. Deprived neighbourhoods have generally greater numbers of fast-food outlets [16, 38], and there is evidence that the influence of neighbourhood environments vary by educational attainment as a marker of SEP [39]. However, there has been little research on the interaction between fast-food access and household income, which may hold implications for diet and weight via different mechanisms. Low-income consumers, in particular, may be disproportionately affected by the presence of fast-food outlets [14], which serve large portions of energy-dense, calorific foods at low prices [40].

With mounting evidence of the adverse influence of fast-food outlets on health and the abundance of fast-food outlets in deprived areas, the proliferation of these outlets has become a public health concern. Policies are now in place in many regions of the UK to stem growth in this retail sector [41], while in the US, a moratorium was placed on the opening of new fast-food outlets in South Los Angeles, for example [42]. At the same time, more empirical research is needed to better understand the magnitude of influence of fast-food outlets on health and their contribution to social inequalities.

Using a large sample from the UK Biobank, the aim of this study was to establish the independent and combined associations (including interactions) of each of neighbourhood fast-food access and household income, with processed meat consumption and multiple measures of adiposity.

## Methods

### Study sample

UK Biobank is a cross-sectional, observational cohort study, which recruited 502,656 people between 2006 and 2010. Those registered with the National Health Service and living within 25 miles of the 22 UK assessment centres were invited to participate in the study. The UK Biobank study design has been reported in detail previously [43]. UK Biobank received ethical approval from the North West Multi-centre Research Ethics Committee (MREC), the Community Health Index Advisory Group (CHIAG) and the Patient Information Advisory Group (PIAG).

### Exposure – Neighbourhood fast-food outlet proportion and household income

For participants attending the three UK Biobank Greater London assessment centres of Barts, Hounslow and Croydon (*n* = 68,850) [44], neighbourhood food outlet exposure metrics were derived in 2014 using 2012 UKMap data courtesy of The GeoInformation Group [44]. UKMap data are collected for Greater London via field survey, are updated twice per year, and have a stated spatial accuracy of ±1 m. Numbers of outlets by type were summed within a 1 mile straight-line radius (circular) ‘neighbourhood’ buffer of participants’ geocoded home street address, a distance equating to a 15-min walk for an average adult and previously linked to actual food shopping behaviours [45]. Fast-food outlets were described in UKMap data as ‘take-away’ outlets, defined as outlets selling food and/or drink for consumption off the premises (excluding general and convenience stores, coffee shops and supermarkets). We expressed the number of neighbourhood fast-food outlets as a proportion (%) of all neighbourhood food outlets (sum of counts of fast-food outlets, supermarkets, restaurants, convenience stores, cafes, and specialist stores). Household income (£/year) was self-reported by UK Biobank participants using the following income brackets: <£31,000, £31,000-£51,999, £52,000-£100,000, >£100,000.

### Outcomes – Processed meat consumption and adiposity

Participants completed a dietary screener, which included questions on the consumption frequency of a limited range of foods from major food groups, as part of a questionnaire relating to their general lifestyle during baseline visits to UK Biobank assessment centres. Processed meat consumption (defined by UK Biobank as consumption of foods such as bacon, ham, sausage, meat pies, kebabs, burgers, chicken nuggets) served as our primary dietary outcome, as a proxy for fast-food consumption, because this type of food is commonly available in fast-food outlets [32, 46, 47]. Processed meat consumption frequency was measured by UK Biobank as follows: “How often [do you] eat processed meats…?”, with six possible response options from “Never” to “Once or more daily”. For this analysis, frequencies were dichotomised, with those reporting more than once per week defined as frequent processed meat consumers. Consumption of fast-food approximately once per week has been associated with cardio-metabolic risk [6], akin to the risk associated with frequent processed meat consumption [3–6], as well as with increased body weight over time [33]. Nearly a quarter of UK adults consume fast-foods at home at least weekly [48]. Body mass index (BMI, kg/m^2^) was calculated from measured height and weight, with participants having a BMI ≥ 30 classified as obese. Body fat (%) was measured using bioelectrical impedance analysis, with the Tanita BC418MA body composition analyser (Tanita, Amsterdam).

### Statistical analysis

We used multivariable linear and binomial logistic regression models to examine associations of proportion of fast-food outlets in the home neighbourhood and household income, in relation to BMI, percentage body fat, odds of frequent processed meat consumption and obesity. We also calculated adjusted risk ratios (RRs) for obesity and processed meat consumption outcomes, which are presented in Additional files. We tested for interaction of fast-food outlet proportion (quartiles) and household income (four groups) on a multiplicative scale using an F-test for linear models and a likelihood ratio test for logistic models.

We also tested for interaction on an additive scale using relative excess risk due to interaction (RERI). Following STROBE reporting guidelines [49] we estimated the separate and combined associations of neighbourhood fast-food outlet proportion and household income on the odds of being obese, and of frequent processed meat consumption using binomial logistic regressions with a single reference category. RERI was calculated using odds ratios (ORs) as follows:

RERI = OR_11_ – OR_10_ – OR_01_ + 1

where ORs are for being obese or a frequent consumer of processed meat, for those with lowest incomes and most-exposed to fast-food outlets (OR_11_), those with lowest incomes and least-exposed (OR_10_) and those with highest incomes and most-exposed (OR_01_) [49, 50]. RERI scores > 0 suggest positive interaction, or a greater risk due to interaction than would be attributable to the additive effects of each of these factors in isolation [49].

Adjusted models included the following covariates, also reported in the UK Biobank lifestyle questionnaire: age, sex, ethnicity, smoking status, number of household residents, highest educational attainment (five categories), UK Biobank assessment centre attended, and the sum of counts of supermarkets, restaurants, convenience stores, cafes and specialist stores within 1 mile Euclidean (straight-line) radius buffers of home address. To establish independent associations, both household income and fast-food proportion models were mutually adjusted.

This was a complete case analysis, with the Greater London UK Biobank sample restricted to those with complete data across all outcomes and covariates of interest (Additional file 1). Exclusions resulted in final sample sizes for BMI, processed meat consumption and percentage body fat models of 51,361, 51,090 and 50,766, respectively. Analytic samples remained representative of the UK Biobank participants attending assessment centres in Greater London across key variables (Additional file 2). Participant data were collected 2006–2010 and analysed in 2016 using Stata 14 (StataCorp LP., Texas).

## Results

### Sample characteristics

Descriptive statistics, overall and stratified by quartiles of neighbourhood fast-food outlet proportion, are shown in Table 1 (and also stratified by household income in Additional file 3). The sample had a mean age of 56 years (aged 38–72 years overall), with 44% of participants’ men and approximately 80% identifying their ethnicity as white. Mean BMI and body fat were 26.9 kg/m^2^ and 30.8% respectively, with 21.8% obese, and 27.7% of the sample consuming processed meat more than once per week. Fast-food outlets constituted 18.4% of neighbourhood food retail on average, corresponding to a mean of 39.2 fast-food outlets. There were systematic differences in demographic and other variables across fast-food exposure groups. In particular, participants in higher exposure groups were less likely to be white, reported lower incomes and fewer educational qualifications. For the quarter of participants most-exposed to fast-food outlets, 24–45% of neighbourhood food vendors were fast-food outlets. Participants with greatest fast-food exposure tended to have higher BMI, higher intake of processed meat and were more likely to be obese.

**Table 1:** Characteristics of participants in the UK Biobank sample, UK (*n* = 51,361), overall and stratified by quartiles of fast-food outlet proportion

	Quartiles of fast-food outlet proportion				
	Q1 (0–13%)	Q2 (13–17%)	Q3 (17–24%)	Q4 (24–45%)	All
Age, years	56.5 (8.2)	55.7 (8.2)	55.6 (8.2)	56.0 (8.2)	56.0 (8.2)
Men (no. (%))	5552 (43.2)	5621 (43.8)	5709 (44.5)	5643 (43.9)	22,525 (43.9)
Ethnicity (no. (%))					
White	11,430 (89.0)	10,496 (81.8)	9601 (74.8)	9405 (73.2)	40,932 (79.7)
Asian or Asian British	359 (2.8)	966 (7.5)	1322 (10.3)	1187 (9.2)	3834 (7.5)
Black or Black British	350 (2.7)	612 (4.8)	1028 (8.0)	1369 (10.7)	3359 (6.5)
Other	351 (2.7)	402 (3.1)	483 (3.8)	448 (3.5)	1684 (3.3)
Don’t know or Prefer not to say	84 (0.6)	96 (0.7)	124 (1.0)	102 (0.8)	406 (0.8)
Household income, £/year (no. (%))					
< 31,000	3379 (26.3)	3859 (30.1)	4563 (35.5)	4798 (37.4)	16,599 (32.3)
31,000 – 51,999	2396 (18.7)	2657 (20.7)	2756 (21.5)	2881 (22.4)	10,690 (20.8)
52,000 – 100,000	3057 (23.8)	3034 (23.6)	2563 (20.0)	2415 (18.8)	11,069 (21.6)
> 100,000	2409 (18.8)	1504 (11.7)	876 (6.8)	591 (4.6)	5380 (10.5)
Don’t know or Prefer not to say	1606 (12.5)	1779 (13.9)	2083 (16.2)	2155 (16.8)	7623 (14.8)
Highest education (no. (%))					
Compulsory (≤11 y of education) or Other^a^	2074 (16.1)	2703 (21.1)	3675 (28.6)	4286 (33.4)	12,738 (24.8)
Further (12–13 y of education)	1680 (13.1)	1779 (13.9)	2039 (15.9)	2224 (17.3)	7722 (15.0)
Higher (> 13 y of education)	8956 (69.7)	8151 (63.5)	6865 (53.5)	6085 (47.4)	30,057 (58.5)
Prefer not to say	137 (1.1)	200 (1.6)	262 (2.0)	245 (1.9)	844 (1.6)
Current or ex-smoker (no. (%))	6304 (49.1)	6109 (47.6)	5759 (44.8)	5587 (43.5)	23,759 (46.3)
Anthropometric and Diet Outcomes					
BMI, kg/m^2^	26.1 (4.5)	26.6 (4.8)	27.3 (5.0)	27.6 (5.0)	26.9 (4.9)
Body Fat, %	29.8 (8.3)	30.4 (8.4)	31.3 (8.7)	32.0 (8.7)	30.8 (8.6)
Obese, BMI ≥ 30 (no. (%))	2102 (16.4)	2564 (20.0)	3136 (24.4)	3401 (26.5)	11,203 (21.8)
Frequent processed meat consumption^e^	3239 (25.2)	3464 (27.0)	3647 (28.4)	3885 (30.3)	14,235 (27.7)
Food Environment Exposures^b^					
Fast-food outlets	35.7 (30.3)	46.5 (30.2)	40.1 (27.2)	34.5 (16.9)	39.2 (27.1)
Other food outlets^c^	400.6 (422.0)	266.1 (177.0)	161.6 (114.7)	84.9 (46.5)	228.3 (265.0)
Fast-food outlet proportion, %^d^	9.1 (2.9)	15.0 (1.2)	20.1 (2.1)	29.4 (3.5)	18.4 (7.9)

Data are mean (standard deviation) unless otherwise stated; percentages represent column percentage | ^a^ Those reporting ‘Other’ education will include those with no and non-British qualifications | ^b^ Counts of food outlets within 1 mile Euclidean (straight line) radius buffers of home address | ^c^ Sum of counts of Supermarkets, Restaurants, Convenience stores, Cafes and Specialist stores | ^d^ Fast-food outlets expressed as a proportion of the sum of counts of Fast-food outlets, Supermarkets, Restaurants, Convenience Stores, Cafes and Specialist stores | ^e^ Frequent consumption was defined as more than once per week; processed meat includes bacon, ham, sausages, meat pies, kebabs, burgers, chicken nuggets

### Associations of neighbourhood fast-food outlet proportion with BMI, obesity and frequent processed meat consumption

Greater BMI, percentage body fat, odds of obesity, and frequent processed meat consumption, were each positively associated with a higher proportion of fast-food outlets in neighbourhoods. In the unadjusted model 1, those with the highest proportion of fast-food outlets (Q4) were 1.54 kg/m^2^ heavier (95% CI: 1.42, 1.66; *p* < 0.001) than those with the lowest proportion (Table 2), and had 2.17% higher (95% CI: 1.96, 2.38; *p* < 0.001) body fat (Additional file 4). Adjusting for additional individual-level demographic (model 2), socioeconomic (model 3) and other neighbourhood-level covariates (model 4) attenuated this association. However, those with the highest proportion of fast-food outlets remained on average 0.99 kg/m^2^ heavier (95% CI: 0.85, 1.14; *p* < 0.001) than those with the lowest proportion (Table 2) and had 1.37% higher (95% CI: 1.17, 1.56; *p* < 0.001) body fat (Additional file 4) in our most adjusted models, and with a dose-response association at least across the first three quartiles.

**Table 2:** Associations of quartiles of fast-food outlet proportion with body mass index (estimated using a multivariable linear regression model, *n* = 51,361), obesity (estimated using a binomial logistic regression model *n* = 51,361), and frequent consumption of processed meat (estimated using a binomial logistic regression model, *n* = 51,090) in the Greater London UK Biobank sample

		Model 1^a^		Model 2^b^		Model 3^c^		Model 4^d^	
	Quartile^e^	β	95% CI	β	95% CI	β	95% CI	β	95% CI
Difference in BMI (kg/m^2^)	Q1 (0.0–12.7%)	ref		ref		ref		ref	
	Q2 (12.7–16.9%)	0.53**	0.41, 0.65	0.48**	0.36, 0.59	0.37**	0.25, 0.49	0.35**	0.23, 0.47
	Q3 (16.9–23.7%)	1.21**	1.09, 1.33	1.04**	0.93, 1.16	0.83**	0.70, 0.95	0.80**	0.67, 0.93
	Q4 (23.7–44.6%)	1.54**	1.42, 1.66	1.30**	1.18; 1.42	1.03**	0.89; 1.17	0.99**	0.85, 1.14
		Model 1^a^		Model 2^b^		Model 3^c^		Model 4^d^	
		OR	95% CI	OR	95% CI	OR	95% CI	OR	95% CI
Odds of obesity (BMI ≥ 30 kg/m^2^)	Q1 (0.0–12.7%)	ref		ref		ref		ref	
	Q2 (12.7–16.9%)	1.28**	1.20, 1.36	1.26**	1.18, 1.34	1.20**	1.12, 1.28	1.19**	1.11, 1.27
	Q3 (16.9–23.7%)	1.65**	1.55, 1.76	1.57**	1.48, 1.67	1.43**	1.34, 1.53	1.42**	1.32, 1.52
	Q4 (23.7–44.6%)	1.84**	1.73, 1.96	1.70**	1.60, 1.81	1.53**	1.43, 1.65	1.51**	1.40, 1.64
		Model 1^a^		Model 2^f^		Model 3^c^		Model 4^d^	
		OR	95% CI	OR	95% CI	OR	95% CI	OR	95% CI
Odds of frequent consumption of processed meat^g^ (> once per week)	Q1 (0.0–12.7%)	ref		ref		ref		ref	
	Q2 (12.7–16.9%)	1.10*	1.04, 1.16	1.15**	1.08, 1.22	1.10*	1.04, 1.17	1.10*	1.03, 1.16
	Q3 (16.9–23.7%)	1.18**	1.12, 1.25	1.29**	1.22, 1.36	1.19**	1.12, 1.27	1.18**	1.11, 1.26
	Q4 (23.7–44.6%)	1.29**	1.22, 1.37	1.45**	1.36, 1.53	1.30**	1.22, 1.39	1.28**	1.19, 1.38

*p < 0.05; ** *p* < 0.001 | ^a^ Model 1 is an unadjusted model | ^b^ Model 2 adjusts for age, sex, ethnicity, smoking status | ^c^ Model 3 additionally adjusts for household income, number in household, highest educational attainment and UK Biobank assessment centre attended | ^d^ Model 4 additionally adjusts for sum of counts of Supermarkets, Restaurants, Convenience stores, Cafes and Specialist Stores within 1 mile Euclidean (straight line) radius buffers of home address | ^e^ Q1 = quartile with lowest fast-food outlet proportion in home neighbourhood (min-max %) – Q4 = quartile with greatest fast-food outlet proportion in home neighbourhood (min-max %) | ^f^ Model 2 adjusts for age, sex, ethnicity | ^g^ Includes bacon, ham, sausages, meat pies, kebabs, burgers, chicken nuggets

In our unadjusted binomial logistic regression model (Table 2), those with the highest proportion of fast-food outlets had 1.84 (95% CI: 1.73, 1.96) greater odds of being obese than not being obese. These associations were attenuated but remained significant after adjustment for potential confounders (models 2–4); in model 4 those with the highest proportion of fast-food outlets had 1.51 (95% CI: 1.40, 1.64) greater odds of being obese, again with a dose-response association at least across the first three quartiles.

Those with the highest proportion of fast-food outlets had 1.28 (95% CI: 1.19, 1.38) greater odds of being frequent processed meat consumers, relative to those with the lowest proportion. Corresponding risk ratios (RRs) for risk of obesity and frequent processed meat consumption related to fast-food proportion were similar in magnitude and again showed evidence of a dose-response association, and are shown in Additional file 5.

### Associations of household income with BMI, obesity and frequent processed meat consumption

Greater BMI, percentage body fat, odds of obesity, and frequent processed meat consumption, were each systematically associated with lower household income. In unadjusted models, those with lowest household incomes (<£31,000) were 1.73 kg/m^2^ heavier (95% CI: 1.58, 1.88; *p* < 0.001) than those with highest incomes (Table 3); had 3.78% higher (95% CI: 3.52, 4.05; *p* < 0.001) body fat (Additional file 6); and had 2.29 (95% CI: 2.10, 2.49) greater odds of being obese. Significant associations were not observed with processed meat consumption.

**Table 3:** Associations of household income with body mass index (estimated using a multivariable linear regression model, *n* = 51,361), obesity (estimated using a binomial logistic regression model, *n* = 51,361), and frequent consumption of processed meat (estimated using a binomial logistic regression model, *n* = 51,090) in the Greater London UK Biobank sample

	Household Income (£/year)	Model 1^a^		Model 2^b^		Model 3^c^		Model 4^d^	
		β	95% CI	β	95% CI	β	95% CI	β	95% CI
Difference in BMI (kg/m^2^)	> 100,000	ref		ref		ref		ref	
	52,000–100,000	0.71**	0.55, 0.87	0.60**	0.45, 0.76	0.44**	0.29, 0.60	0.33**	0.17, 0.48
	31,000–51,999	1.17**	1.01, 1.33	0.95**	0.80, 1.11	0.67**	0.51, 0.83	0.51**	0.35, 0.67
	< 31,000	1.73**	1.58, 1.88	1.36**	1.21, 1.51	0.85**	0.69, 1.01	0.68**	0.52, 0.84
		Model 1^a^		Model 2^b^		Model 3^c^		Model 4^d^	
		OR	95% CI	OR	95% CI	OR	95% CI	OR	95% CI
Odds of obesity (BMI ≥ 30 kg/m^2^)	> 100,000	ref		ref		ref		ref	
	52,000–100,000	1.42**	1.29, 1.56	1.37**	1.24, 1.50	1.29**	1.17, 1.41	1.22**	1.11, 1.34
	31,000–51,999	1.70**	1.55, 1.86	1.57**	1.43, 1.72	1.41**	1.28, 1.54	1.32**	1.20, 1.45
	< 31,000	2.29**	2.10, 2.49	2.02**	1.84, 2.20	1.66**	1.51, 1.82	1.54**	1.41, 1.69
		Model 1^a^		Model 2^e^		Model 3^c^		Model 4^d^	
		OR	95% CI	OR	95% CI	OR	95% CI	OR	95% CI
Odds of frequent consumption of processed meat^f^ (> once per week)	> 100,000	ref		ref		ref		ref	
	52,000–100,000	1.08*	1.00, 1.16	1.17**	1.09, 1.27	1.13*	1.05, 1.22	1.10*	1.02, 1.19
	31,000–51,999	1.01	0.94, 1.09	1.24**	1.15, 1.34	1.19**	1.10, 1.29	1.14*	1.06, 1.24
	< 31,000	1.01	0.94, 1.08	1.38**	1.28, 1.49	1.30**	1.20, 1.41	1.25**	1.15, 1.35

Measure of interaction (fast-food exposure*household income with difference in BMI) on a multiplicative scale based on an F-test, *P* = 0.230 | *p < 0.05; ** *p* < 0.001 | ^a^ Model 1 adjusts for number in household | ^b^ Model 2 additionally adjusts for age, sex, ethnicity, smoking status | ^c^ Model 3 additionally adjusts for highest educational attainment and UK Biobank assessment centre attended | ^d^ Model 4 additionally adjusts for fast-food outlet proportion and the sum of counts of Supermarkets, Restaurants, Convenience stores, Cafes and Specialist Stores within 1 mile Euclidean (straight line) radius buffers of home address | ^e^ Model 2 adjusts for age, sex, ethnicity | ^f^ Includes bacon, ham, sausages, meat pies, kebabs, burgers, chicken nuggets

Additional covariate adjustment attenuated these associations; however, they remained significant. In our maximally-adjusted models, those with lowest incomes were 0.68 kg/m^2^ heavier (95% CI: 0.52, 0.84; *p* < 0.001), had 0.83% higher body fat, had 1.54 (95% CI: 1.41, 1.69) greater odds of being obese, and 1.25 (95% CI: 1.15, 1.35) greater odds of frequent processed meat consumption, compared to those with highest incomes. There was no evidence of interaction on a multiplicative scale between fast-food outlet proportion and household income on BMI (*P* = 0.230). Corresponding RRs for risk of obesity and frequent processed meat consumption related to household income are shown in Additional file 7.

### Combined associations of neighbourhood fast-food outlet proportion and household income with each of obesity and frequent processed meat consumption

There was no evidence of interaction on a multiplicative scale between fast-food outlet proportion and household income on obesity (*P* = 0.054) and frequent processed meat consumption (*P* = 0.725). We observed evidence of interaction on an additive scale using RERI, which for obesity was 0.03 and for frequent processed meat consumption was 0.18. This demonstrated that greater odds of obesity and frequent processed meat consumption were associated with highest fast-food exposure and lowest household income in combination, in excess of the odds associated with either highest fast-food outlet exposure or lowest household income in isolation. Table 4 shows adjusted binomial logistic regression results for each combination of fast-food exposure quartile and level of household income on odds of obesity. Those with the highest proportion of fast-food outlets and lowest income had 2.43 (95% CI: 2.09, 2.84) greater odds of being obese, relative to those with the lowest proportion of fast-food outlets and highest household incomes.

**Table 4:** Additive interaction between quartiles of fast-food outlet proportion and household income on the odds of being obese (BMI ≥ 30 kg/m^2^), modelled using a binomial logistic regression model in the UK Biobank sample, UK (*n* = 51,361)

	Quartiles of fast-food outlet proportion^a^								Fast-food outlet exposure (Q4) within household income strata
	Q1 (0–13%)		Q2 (13–17%)		Q3 (17–24%)		Q4 (24–45%)		
Household Income (£/year)	Obese/not obese (n)	OR (95% CI)	Obese/not obese (n)	OR (95% CI)	Obese/not obese (n)	OR (95% CI)	Obese/not obese (n)	OR (95% CI)	
> 100,000	264/2145	ref^b^	206/1298	1.30 (1.07, 1.59); *P* = 0.008^c^	143/733	1.60 (1.27, 2.00); *P* < 0.001^c^	104/487	1.73 (1.34, 2.23); *P* < 0.001^c^	1.53 (1.12, 2.08) *P* = 0.008^d^
52,000–100,000	433/2624	1.27 (1.08, 1.50); *P* = 0.004^c^	475/2559	1.44 (1.22, 1.69); *P* < 0.001^c^	494/2069	1.81 (1.53, 2.13); *P* < 0.001^c^	581/1834	2.26 (1.91, 2.66); *P* < 0.001^c^	1.69 (1.43, 2.02) *P* < 0.001^d^
31,000–51,999	389/2007	1.42 (1.20, 1.69); *P* < 0.001^c^	495/2162	1.64 (1.39, 1.93); *P* < 0.001^c^	605/2151	1.95 (1.66, 2.29); *P* < 0.001^c^	724/2157	2.20 (1.87, 2.58); *P* < 0.001^c^	1.51 (1.27, 1.80) *P* < 0.001^d^
< 31,000	687/26922	1.67 (1.43, 1.95); *P* < 0.001^c^	957/2902	2.05 (1.76, 2.39); *P* < 0.001^c^	1271/3292	2.30 (1.98, 2.68); *P* < 0.001^c^	1401/3397	2.43 (2.09, 2.84); *P* < 0.001^c^	1.56 (1.37, 1.78) *P* < 0.001^d^
Household income (< £31,000/year) within takeaway exposure strata		1.58 (1.34, 1.88) *P* < 0.001^e^		1.36 (1.15, 1.65) *P* = 0.001^e^		1.49 (1.21, 1.82) *P* < 0.001^e^		1.53 (1.22, 1.93) *P* < 0.001^e^	

Measure of interaction (fast-food exposure*household income with odds of obesity) on a multiplicative scale based on a likelihood ratio test, *P* = 0.054 | Measure of interaction on an additive scale for Q4 fast-food exposure and household income < £31,000 (RERI) = 0.03. RERI scores > 0 suggest positive interaction and departure from additivity | ORs are adjusted for age, sex, ethnicity smoking status, number in household, highest education attainment, UK Biobank assessment centre attended, and the sum of counts of Supermarkets, Restaurants, Convenience stores, Cafes and Specialist Stores within 1 mile Euclidean (straight line) radius buffers of home address | ^a^ Q1 = quartile with lowest fast-food outlet proportion in home neighbourhood (min – max %) – Q4 = quartile with greatest fast-food outlet proportion in home neighbourhood (% range) | ^b^ Single reference group; those exposed to the highest proportion of fast-food outlets and with the highest household income | ^c^ ORs and *p* values relative to single reference group (ref) | ^d^ ORs and *P* values relative to those who were least exposed to fast-food outlets within strata of household income | ^e^ ORs and *P* values relative to those with the highest household incomes within strata of fast-food outlet exposure

Table 5 shows adjusted binomial logistic regression results for each combination of fast-food outlet proportion quartile and level of household income on odds of frequent processed meat consumption. Those with the highest proportion of fast-food outlets and lowest income had 1.46 (95% CI: 1.29, 1.65) greater odds of consuming processed meats frequently, relative to those with the lowest proportion of fast-food outlets and highest household incomes.

**Table 5:** Additive interaction between quartiles of fast-food outlet proportion and household income on the odds of frequent processed meat consumption^a^ (more than once per week), modelled using a binomial logistic regression model in the Greater London UK Biobank sample, UK (*n* = 51,090)

	Quartiles of fast-food outlet proportion^b^								Fast-food outlet exposure (Q4) within household income strata
	Q1 (0–13%)		Q2 (13–17%)		Q3 (17–24%)		Q4 (24–45%)		
Household Income (£/year)	More than once/once or less (n)	OR (95% CI)	More than once/once or less (n)	OR (95% CI)	More than once/once or less (n)	OR (95% CI)	More than once/once or less (n)	OR (95% CI)	
> 100,000	653/1756	ref ^c^	401/1103	0.95 (0.82, 1.10); *P* = 0.495^d^	253/623	1.06 (0.89, 1.27); *P* = 0.502^d^	177/414	1.11 (0.90, 1.37); *P* = 0.335^d^	1.19 (0.93, 1.54) *P* = 0.158^e^
52,000–100,000	782/2274	0.98 (0.86, 1.11); *P* = 0.742^d^	860/2172	1.13 (1.00, 1.29); *P* = 0.047^d^	788/1769	1.22 (1.07, 1.39); *P* = 0.003^d^	790/1622	1.34 (1.17, 1.54); *P* < 0.001^d^	1.39 (1.20, 1.62) *P* < 0.001^e^
31,000–51,999	593/1798	1.06 (0.92, 1.21); *P* = 0.418^d^	695/1960	1.12 (0.99, 1.28); *P* = 0.079^d^	785/1963	1.25 (1.10, 1.42); *P* = 0.001^d^	902/1975	1.41 (1.24, 1.61); *P* < 0.001^d^	1.31 (1.12, 1.54) *P* = 0.001^e^
< 31,000	842/2526	1.17 (1.03, 1.33); *P* = 0.015^d^	1036/2799	1.29 (1.14, 1.46); *P* < 0.001^d^	1277/3253	1.38 (1.22, 1.55); *P* < 0.001^d^	1423/3350	1.46 (1.29, 1.65); *P* < 0.001^d^	1.29 (1.13, 1.46) *P* < 0.001^e^
Household income (< £31,000/year) within takeaway exposure strata		1.11 (0.97, 1.28) *P* = 0.117^f^		1.27 (1.09, 1.48) *P* = 0.002^f^		1.30 (1.09, 1.56) *P* = 0.003^f^		1.45 (1.18, 1.78) *P* < 0.001^f^	

Measure of interaction (fast-food exposure*household income with odds of processed meat consumption) on a multiplicative scale based on a likelihood ratio test, *P* = 0.725 | Measure of interaction on an additive scale for Q4 fast-food exposure and household income < £31,000 (RERI) = 0.18. RERI scores > 0 suggest positive interaction and departure from additivity | ORs are adjusted for age, sex, ethnicity smoking status, number in household, highest education attainment, UK Biobank assessment centre attended, and the sum of counts of Supermarkets, Restaurants, Convenience stores, Cafes and Specialist Stores within 1 mile Euclidean (straight line) radius buffers of home address | ^a^ Includes bacon, ham, sausages, meat pies, kebabs, burgers, chicken nuggets | ^b^ Q1 = quartile with lowest fast-food outlet proportion in home neighbourhood (min – max %) – Q4 = quartile with greatest fast-food outlet proportion in home neighbourhood (% range) | ^c^ Single reference group; those exposed to the highest proportion of fast-food outlets and with the highest household income | ^d^ ORs and *p* values relative to single reference group (ref) | ^e^ ORs and *P* values relative to those who were least exposed to fast-food outlets within strata of household income | ^f^ ORs and *P* values relative to those with the highest household incomes within strata of fast-food outlet exposure

## Discussion

In a large UK adult sample, our results showed clear, consistent associations between neighbourhood fast-food outlet proportion and processed meat consumption, as well as proportion in relation to multiple objective measures of adiposity (body mass index and body fat percentage), including odds of obesity, and with some evidence of dose-response observed. We also showed independent associations between household income and each of these outcomes. We found no evidence of multiplicative interaction, suggesting that associations between fast-food proportion and our outcomes were not significantly different across household income groups. However, we demonstrated the magnitude of obesity and frequent processed meat consumption odds within population-subgroups, including the marginally excess odds (evidence of additive interaction) associated with both highest fast-food outlet proportion and lowest income for these outcomes. This double burden of individual-level disadvantage and neighbourhood-level imbalance towards fast-food retail, holds clear implications for public health and understanding the generation and persistence of social inequalities in diet, health and NCD risk [9, 10].

Broadly, we were able to demonstrate a clear relationship between neighbourhood fast-food outlet exposure, diet and body weight, thereby making an important contribution to an equivocal evidence base. Where comparisons can be drawn, the relationships we observed with fast-food exposure were consistent with those of recent research, for the outcomes of unhealthy diet [19, 20, 31, 39, 51], and obesity [18, 19, 24, 28, 30, 31, 39], especially those using a relative measure of fast-food access [20, 24, 25, 28, 30]. A previous UK study observed for those most-exposed to fast-food outlets (relative to those least-exposed) a 0.97 kg/m^2^ difference in BMI (our study, β = 0.99 kg/m^2^) and an odds of 2.15 for obesity (our study, OR = 1.51) [31]. Another showed consistent associations, of a 0.90 kg/m^2^ difference in BMI across combined home and work neighbourhood fast-food exposure [39]. While a recent study that also used UK Biobank data found comparatively weaker and less consistent associations between fast-food access and multiple measures of adiposity [52], these differences may be explained to some extent by methodological dissimilarities [53]. Mason et al. used an absolute measure of street network proximity to the nearest fast-food outlet, precluding adjustment for wider food environment context, as facilitated through our use of a relative measure of fast-food density. It has been suggested that methodological inconsistencies might explain divergent findings across other recent studies [18, 19, 54–56], examples of which may include: use of proximity as opposed to density; different delineations of neighbourhood extent; use of area- as opposed to person-based exposure measures; and multiple possible aspects of inaccuracy in underpinning food outlet data [57]. Elsewhere, only one previous study has attempted to establish the magnitude of the combined associations of neighbourhood fast-food access and individual-level SEP, with unhealthy diet and adiposity. That study found highest fast-food exposure combined with lowest education to result in a 3.12 greater odds of obesity, relative to those least-exposed and most-educated [39]. In our study, the combination of highest fast-food exposure and lowest income was associated with a 2.43 greater odds of obesity, and a 1.46 greater odds of frequent processed meat consumption, relative to those least-exposed and with highest incomes.

In this sub-group (those most exposed and with lowest incomes) we also observed marginally excess odds of both obesity and frequent processed meat consumption, over and above the additive effects of each risk factor in isolation, which is evidence of additive interaction. We observed no evidence of a differential impact of fast-food exposure across household income levels (multiplicative interaction), in contrast to a US study, which showed that neighbourhood fast-food access was only related to BMI among low income adults [58]. However, the relative merits of assessing interaction on additive vs multiplicative scales has long been the subject of epidemiological debate. While analysis of multiplicative interactions is more commonplace, it has been suggested that additive interaction bears particular relevance to public health, for which a key concern is the risk of disease in the proportion of the population for whom the risk factors occur together [59, 60].

### Implications for public health

These results suggest that those experiencing a double of burden of lowest SEP and greatest fast-food exposure, are especially at risk of frequently consuming processed meats and being obese. The mechanisms underpinning these relationships are likely to include the low cost [40] and perceived value for money of fast food [61], appealing to the price sensitivity of low-income consumers [14], and thereby influencing how and how frequently these outlets are used. We argue that this compounding of individual-level disadvantage by neighbourhood-level high proportion of fast-food outlets, is implicated in social inequalities in diet, health and NCD risk [9, 10].

In this context, the greater number of fast-food outlets commonly found in deprived areas [16], and the relative concentration of fast-food outlets in these areas over the last decade in developed countries [38], is cause for deepening public health concern [16, 62]. However, our findings highlight the potential for upstream, neighbourhood-level dietary public health interventions. Not only to improve population-level diet and health [63], but those of at risk population sub-groups in particular [64, 65], which may contribute to the levelling of social inequalities in health. Such interventions, which include exclusion zones around schools or other planning regulations designed to reduce clustering and proliferation of new fast-food businesses, have already begun to be implemented across the US and the UK [41, 42, 66], including boroughs of Greater London where this research took place [67]. For example, these restrictions form an important part of the Mayor of London’s *London Plan* [68], which includes a focus on healthy food environments in general, and access to hot food takeaways specifically, up to the year 2041.

### Limitations and strengths

Our study has a number of limitations, foremost of which is the cross-sectional, observational study design, limiting causal inference. We employed a theoretically- and behaviourally-relevant definition of residential ‘neighbourhood’ [45], which also has precedent for use [31, 39] however which may not match the neighbourhood perceptions or align with the food purchasing behaviours of individuals in this study, and which may have resulted in exposure misclassification. While residential location constitutes an important daily anchor point [69], ensuring relevance of assessing neighbourhood food outlet exposure around this location, we could not assess exposure to the food environment within wider activity spaces [70]. That said, research has shown that magnitude of non-residential neighbourhood food outlet exposure is unrelated to that of residential exposure [71, 72], resulting in random error (not bias) that would have likely served to attenuate our parameter estimates towards the null [73]. UK Biobank data was collected 2006–2010, with neighbourhood food exposure metrics derived from 2012 UKMap data [44]. This resulted in temporal mismatch, although grouping exposure estimates into quarters is likely to have reduced the impact of misclassification. Further misclassification may have emerged from use of UKMap data, which is of unknown completeness, including how this may vary geographically.

We used frequency of processed meat consumption as a proxy for fast-food intake, however, while fast-food outlets are likely to be an important source of these types of foods, such processed foods could be obtained from non-fast-food outlets [74]. Moreover, UK Biobank had only limited detail of dietary intakes, with no information on total diet, energy intake or portion sizes of food and drink consumed. Thus we could estimate only frequency of processed meat consumption, but not total amount. We used household income as our marker of socioeconomic status, in lieu of other markers including educational attainment and occupational social class, which are both imperfectly correlated with income [75]. Our results may be sensitive to the selection of income as our marker of SEP, however, given that findings were consistent with those of a similar study that used education as a marker of socioeconomic status [39], this appears unlikely. Finally, participants in our analytic sample were located in Greater London. While this may influence generalisability, once again the present results were consistent with those we have reported previously from elsewhere in the UK [31, 39].

These limitations are balanced by a number of strengths. First, the large sample size, allied with socioeconomic heterogeneity, allowed robust sub-group analyses. Second, neighbourhood fast-food outlet access was well-characterised, including accounting for fast-food outlets in the context of wider neighbourhood-level food outlet access [53]. Third, we used two objective measures of adiposity, alongside a dietary outcome, demonstrating consistent and complementary associations. Lastly, our study satisfies a number of Bradford Hill criteria, which are useful for inferring causality – in this case, neighbourhood ‘effects’ - from a cross-sectional study design, including: *consistency* (across multiple epidemiologic studies in different locations and populations, and perhaps to the greatest extent with studies that also employed a relative measure of fast-food exposure), *biological gradient* (evidence of dose-response, especially for adiposity models), and *plausibility* (of a cause-and-effect relationship) [76].

## Conclusions

Our study demonstrated independent associations between each of income and neighbourhood fast-food exposure, with diet and two objectively measured adiposity outcomes, in a large sample of UK adults. Moreover, we provide evidence of the double burden of low income and an unhealthy neighbourhood food environment, resulting in an additive interaction and an excess and substantially greater likelihood of unhealthy diet and obesity. Although further work is necessary, these findings support emerging guidelines regarding the regulation of neighbourhood fast-food access for the promotion of population health.

## Additional files

Additional file 1: Flow diagram for UK Biobank sample restriction, for body weight-, processed meat consumption- and percentage body fat-based analyses reported in this study. (DOCX 37 kb)

Additional file 2: UK Biobank participants attending Greater London assessment centres (Barts, Croydon, Hounslow; *n* = 68,850) and UK Biobank analytic sample (*n* = 51,361) demographic comparisons. (DOCX 20 kb)

Additional file 3: Characteristics of participants in the UK Biobank sample, UK (*n* = 51,361), overall and stratified by household income. (DOCX 24 kb)

Additional file 4: Associations of quartiles of fast-food outlet proportion with body fat percentage (estimated using a multivariable linear regression model, *n* = 50,766) in the Greater London UK Biobank sample. (DOCX 20 kb)

Additional file 5: Adjusted risk ratios (RRs) describing the associations of quartiles of fast-food outlet proportion with body mass index (estimated using a multivariable linear regression model, *n* = 51,361), obesity (estimated using a binomial logistic regression model *n* = 51,361), and frequent consumption of processed meat (estimated using a binomial logistic regression model, *n* = 51,090) in the Greater London UK Biobank sample. (DOCX 22 kb)

Additional file 6: Associations of household income with body fat percentage (estimated using a multivariable linear regression model, *n* = 50,766) in the Greater London UK Biobank sample. (DOCX 20 kb)

Additional file 7: Adjusted risk ratios (RRs) describing the associations of household income with body mass index (estimated using a multivariable linear regression model, *n* = 51,361), obesity (estimated using a binomial logistic regression model, *n* = 51,361), and frequent consumption of processed meat (estimated using a binomial logistic regression model, *n* = 51,090) in the Greater London UK Biobank sample. (DOCX 22 kb)

## Abbreviations

BMI: Body mass index
CHIAG: Community Health Index Advisory Group
MREC: Multi-centre Research Ethics Committee
NCD: Non-communicable disease
OR: Odds ratio
PIAG: Patient Information Advisory Group
Q: Quartile
RERI: Relative excess risk due to interaction
RR: Risk ratio
SEP: Socioeconomic position
STROBE: STrengthening the Reporting of Observational studies in Epidemiology
UK: United Kingdom
US: United States
WHO: World Health Organization

## References

[1] Newton JN, Briggs ADM, Murray CJL, Dicker D, Foreman KJ, Wang H, Naghavi M, Forouzanfar MH, Ohno SL, Barber RM (2015). Changes in health in England, with analysis by English regions and areas of deprivation, 1990-2013: a systematic analysis for the global burden of disease study 2013. Lancet.

[2] Rayner M, Scarborough P (2005). The burden of food related ill health in the UK. J Epidemiol Community Health.

[3] Micha R, Wallace SK, Mozaffarian D (2010). Red and processed meat consumption and risk of incident coronary heart disease, stroke, and diabetes mellitus. Circulation.

[4] Micha R, Penalvo JL, Cudhea F, Imamura F, Rehm CD, Mozzaffarian D (2017). Association between dietary risk factors and mortality from heart disease, stroke, and type 2 diabetes in the United States. JAMA.

[5] Micha R, Wallace SK, Mozzaffarian D (2011). Red and processed meat consumption and risk of incident heart disease, stroke, and diabetes: a systematic review and meta-analysis. Circulation.

[6] Bouvard V, Loomis D, Guyton KZ, Grosse Y, Ghissassi FE, Benbrahim-Tallaa L, Guha N, Mattock H, Straif K (2015). International Agency for Research on Cancer monograph working group.,: Cancinogenicity of consumption of red and processed meat. Lancet Oncol.

[7] Mills Susanna, Adams Jean, Wrieden Wendy, White Martin, Brown Heather (2018). Sociodemographic characteristics and frequency of consuming home-cooked meals and meals from out-of-home sources: cross-sectional analysis of a population-based cohort study. Public Health Nutrition.

[8] McLaren L (2007). Socioeconomic status and obesity. Epidemiol Rev.

[9] Moody A, Cowley G, Fat LG, Mindell JS (2016). Social inequalities in prevalence of diagnosed and undiagnosed diabetes and impaired glucose regulation in participants in the health surveys for England series. BMJ Open.

[10] Cooper RS (2001). Social inequality, ethnicity and cardiovascular disease. Int J Epidemiol.

[11] Lang T, Caraher M, Dixon P, Carr-Hill R (1999). Cooking skills and health. Book cooking skills and health.

[12] Parmenter K, Waller J, Wardle J (2000). Demographic variation in nutrition knowledge in England. Health Educ Res.

[13] Adams J, White M (2015). Prevalence and socio-demographic correlates of time spent cooking by adults in the 2005 UK time use survey. Cross-sectional analysis. Appetite.

[14] Barton KL, Wrieden WL, Sherriff A, Armstrong J, Anderson AS (2015). Trends in socio-economic inequalities in the Scottish diet: 2001-2009. Public Health Nutr.

[15] Pechey Rachel, Monsivais Pablo (2016). Socioeconomic inequalities in the healthiness of food choices: Exploring the contributions of food expenditures. Preventive Medicine.

[16] Cavill N, Rutter H: Healthy people, healthy places briefing: obesity and the environment: regulating the growth of fast food outlets. In Book Healthy people, healthy places briefing: obesity and the environment: regulating the growth of fast food outlets (editor ed.^eds.). City: Public Health England; 2013.

[17] Jones A, Bentham P, Foster C, Hillsdon M, Panter J (2007). Obesogenic environments: evidence review. Foresight tackling obesities: future choices project long science review. Book obesogenic environments: evidence review. Foresight tackling obesities: future choices project long science review.

[18] Cobb LK, Appel LJ, Franco M, Jones-Smith JC, Nur A, Anderson AM (2015). The relationship of the local food environment with obesity: a systematic review of methods, study quality, and results. Obesity.

[19] Black C, Moon G, Baird J (2013). Dietary inequalities: what is the evidence for the effect of the neighbourhood food environment?. Health Place.

[20] Richardson AS, Meyer KA, Green Howard A, Boone-Heinonen J, Popkin BM, Evenson KR, Shikany JM, Lewis CE, Gordon-Larsen P (2015). Multiple pathways from the neighbourhood food environment to increased body mass index through dietary behaviors: a structural equation-based analysis in the CARDIA study. Health Place.

[21] Luan H, Minaker LM, Law J (2016). Do marginalized neigbourhoods have less healthy retail food environments? An analysis using Bayesian spatial latent factor and hurdle models. Int J Health Geogr.

[22] Cummins S, Clary C, Shareck M (2017). Enduring challenges in estimating the effect of the food environment on obesity. Am J Clin Nutr.

[23] Burgoine T, Mackenbach JD, Lakerveld J, Forouhi NG, Griffin SJ, Brage S, Wareham N, Monsivais P (2017). Interplay of socioeconomic status and supermarket distance in associated with excess obesity risk: a UK cross-sectional study. Int J Environ Res Public Health.

[24] Polsky JY, Moineddin R, Glazier RH, Dunn JR, Booth GL (2016). Relative and absolute availability of fast-food restaurants in relation to the development of diabetes: a population-based cohort study. Canadian Journal of Public Health.

[25] Polsky JY, Moineddin R, Dunn JR, Glazier RH, Booth GL (2016). Absolute and relative densities of fast-food versus other restaurants in relation to weight status: does restaurant mix matter?. Prev Med.

[26] Maguire ER, Burgoine T, Penney T, Monsivais P (2017). Does exposure to the food environment differ by socio-economic position? Comparing area-based and person-centred metrics in the fenland study, UK. Int J Health Geographics.

[27] Clary CM, Ramos Y, Shareck M, Kestens Y (2015). Should we use absolute or relative measures when assessing foodscape exposure in relation to fruit and vegetable intake? Evidence from a wide-scale Canadian study. Prev Med.

[28] Stark JH, Neckerman KM, Lovasi GS, Konty K, Quinn J, Arno P, Viola D, Harris TG, Weiss CC, Bader MDM, Rundle A (2013). Neighbourhood food environments and body mass index among new York City adults. J Epidemiol Community Health.

[29] Mercille G, Richard L, Gauvin L, Kestens Y, Payette H, Daniel M (2012). Comparison of two indices of availability of fruits/vegetable and fast food outlets. J Urban Health.

[30] Feng X, Astell-Burt T, Badland H, Mavoa S, Giles-Corti B (2018). Modest ratios of fast food outlets to supermarkets and green grocers are associated with higher body mass index: longitudinal analysis of a sample of 15,229 Australians aged 45 years and older in the Australian National Liveability Study. Health Place.

[31] Burgoine T, Forouhi NG, Griffin SJ, Wareham NJ, Monsivais P (2014). Associations between exposure to takeaway food outlets, takeaway food consumption, and body weight in Cambridgeshire, UK: population based, cross sectional study. BMJ.

[32] Jaworowska A, Blackham TM, Long R, Taylor C, Ashton M, Stevenson L, Davies IG (2014). Nutritional composition of takeaway food in the UK. Nutr Food Sci.

[33] Duffey KJ, Gordon-Larsen P, Jacobs DR, Williams OD, Popkin BM (2007). Differential associations of fast food and restaurant food consumption with 3-y change in body mass index: the coronary artery risk development in young adults study. Am J Clin Nutr.

[34] Smith KJ, Blizzard L, McNaughton SA, Gall SL, Dwyer T, Venn AJ (2012). Takeaway food consumption and cardio-metabolic risk factors in young adults. Eur J Clin Nutr.

[35] Pereira MA, Kartashov AI, Ebbeling CB, Van Horn L, Slattery ML, Jacobs DR, Ludwig DS (2005). Fast-food habits, weight gain, and insulin resistance (the CARDIA study): 15-year prospective analysis. Lancet.

[36] Penney T, Jones NRV, Adams J, Maguire ER, Burgoine T, Monsivais P (2017). Utilization of away-from-home food establishments, dietary approaches to stop hypertension dietary pattern, and obesity. Am J Prev Med.

[37] Miura K, Turrell G (2014). Reported consumption of takeaway food and its contribution to socioeconomic inequalities in body mass index. Appetite.

[38] Maguire ER, Burgoine T, Monsivais P (2015). Area deprivation and the food environment over time: a repeated cross-sectional study on takeaway outlet density and supermarket presence in Norfolk, UK, 1980-2008. Health Place.

[39] Burgoine T, Forouhi NG, Griffin SJ, Brage S, Wareham NJ, Monsivais P (2016). Does neighborhood fast-food outlet exposure amplify inequalities in diet and obesity? A cross sectional study. Am J Clin Nutr.

[40] Drewnowski A, Specter SE (2004). Poverty and obesity: the role of energy density and energy costs. Am J Clin Nutr.

[41] Tipping the scales: Case studies on the use of planning powers to limit hot food takeaways [https://www.local.gov.uk/sites/default/files/documents/tipping-scales-case-studi-bff.pdf]. Accessed 4 April 2018.

[42] Ordinance no. 180103 [https://clkrep.lacity.org/onlinedocs/2007/07-1658_ord_180103.pdf]. Accessed 6 February 2018.

[43] Allen NE, Sudlow C, Peakman T, Collins R (2014). UK biobank data: come and get it. Sci Transl Med.

[44] Sarkar C, Webster C, Gallacher J (2015). UK biobank urban morphometric platform (UKBUMP) - a nationwide resource for evidence-based healthy city planning and publuc health interventions. Ann GIS.

[45] Smith G, Gidlow C, Davey R, Foster C: What is my walking neighbourhood? A pilot study of English adults' definitions of their local walking neighbourhoods. Int J Behav Nutr Phys Activ 2010, 7:DOI:10.1186/1479-5868-1187-1134. 10.1186/1479-5868-7-34 PMC2873577 20459636

[46] Chicken shops and youth obesity: summary of research findings [https://www.shiftdesign.org.uk/content/uploads/2014/09/SHIFT_Chicken-Shop_Research_.pdf]. Accessed 8 February 2018.

[47] Gateshead Council: Hot food takeaway supplementary planning document. In Book Hot food takeaway supplementary planning document (editor ed.^eds.). City; 2015.

[48] Adams J, Goffe L, Brown T, Lake AA, Summerbell C, White M, Wrieden W, Adamson AJ (2015). Frequency and socio-demographic correlates of eating meals out and take-away meals at home: cross-sectional analysis of the UK national diet and nutrition survey, waves 1-4 (2008-12). Int J Behav Nutr Phys Activ.

[49] Knol MJ, VanderWeele TJ (2012). Recommendations for presenting analyses of effect modification and interaction. Int J Epidemiol.

[50] Andersson T, Alfredsson L, Kallberg H, Zdravkovic S, Ahlbom A (2005). Calculating measures of biological interaction. Eur J Epidemiol.

[51] Bernsdorf KA, Lau CJ, Andreasen AH, Toft U, Lykke M, Glűmer C (2017). Accessibility of fast food outlets is associated with fast food intake. A study in the capital region of Denmark. Health Place.

[52] Mason KE, Pearce N, Cummins S (2018). Associations between fast food and physical activity environments and adiposity in mid-life: cross-sectional, observational evidence from UK biobank. Lancet Public Health.

[53] Monsivais P, Burgoine T (2018). The built environment and obesity in UK biobank: right project, wrong data?. Lancet Public Health.

[54] Mackenbach JD, Charreire H, Glonti K, Bárdos H, Rutter H, Compernolle S, De Bourdeaudhuij I, Nijpels G, Brug J, Oppert J-M, Lakerveld J. Exploring the relation of spatial access to fast food outlets with body weight: a mediation analysis. Environ Behav. 2018:1–30.

[55] Jiao J, Moudon AV, Hurvitz PM, Drewnowski A (2015). Health implications of adults' eating at and living near fast food or quick service restaurants. Nutr Diabetes.

[56] Hobbs M, Griffiths C, Green MA, Jordan H, Saunders J, McKenna J. Neigbourhood typologies and associations with body mass index and obesity: a cross-sectional study. Prev Med. 2017: Volume: 111: 1–7. 10.1016/j.ypmed.2017.11.024 29195761

[57] Charreire H, Casey R, Salze P, Simon C, Chaix B, Banos A, Badariotti D, Weber C, Oppert J-M (2010). Measuring the food environment using geographical information systems: a methodological review. Public Health Nutr.

[58] Reitzel LR, Regan SD, Nguyen N, Cromley EK, Strong LL, Wetter DW, McNeill LH (2014). Density and proximity of fast food restaurants and body mass index among African Americans. Am J Public Health.

[59] Kendler KS, Gardner CO (2010). Interpretation of interactions: guide for the perplexed. Br J Psychiatry.

[60] Rothman KJ, Greenland S, Walker AM (1980). Concepts of interaction. Am J Epidemiol.

[61] Drewnowski A (2009). Obesity, diets, and social inequalities. Nutr Rev.

[62] Davies SC: Annual report of the chief medical officer, surveillance volume, 2012: on the state of the public's health. In Book Annual report of the chief medical officer, surveillance volume, 2012: on the state of the public's health (Editor ed.^eds.). City: Department of Health 2014.

[63] Adams J, Mytton O, White M, Monsivais P (2016). Why are some population interventions for diet and obesity more equitable and effective than others? The role of individual agency. PLOS One.

[64] McGill R, Anwar E, Orton L, Bromley H, Lloyd-Williams F, O'Flaherty M, Taylor-Robinson D, Guzman-Castillo M, Gillespie D, Moreira P (2015). Are interventions to promote healthy eating equally effective for all? Systematic review of socioeconomic inequalities in impact. BMC Public Health.

[65] Ford PB, Dzewaltowski DA (2008). Disparities in obesity prevalence due to variation in the retail food environment: three testable hypotheses. Nutr Rev.

[66] Strategies for encouraging healthier 'out of home' food provision: a toolkit for local councils working with small food businesses [https://www.gov.uk/government/uploads/system/uploads/attachment_data/file/604912/Encouraging_healthier_out_of_home_food_provision_toolkit_for_local_councils.pdf]. Accessed 7 October 2017

[67] Greater London Authority (2012). Takeaways toolkit: tools, interventions and case studies to help local authorities develop a response to the health impacts of fast food takeaways. Book takeaways toolkit: tools, interventions and case studies to help local authorities develop a response to the health impacts of fast food takeaways.

[68] Greater London Authority: The London Plan: the spatial development strategy for Greater London. In Book The London Plan: the spatial development strategy for Greater London (Editor ed.^eds.). City: Greater London Authority https://www.london.gov.uk/sites/default/files/new_london_plan_december_2017.pdf; 2017. Accessed 10 June 2018

[69] Matthews SA, Yang T-C (2013). Spatial polygamy and contextual exposures (SPACEs): promoting activity space approaches in research on place and health. Am Behav Sci.

[70] Kestens Y, Lebel A, Daniel M, Thériault M, Pampalon R (2010). Using experienced activity spaces to measure foodscape exposure. Health Place.

[71] Zenk SN, Schulz AJ, Matthews SA, Odoms-Young A, Wilbur J, Wegrzyn L, Gibbs K, Braunschweig C, Stokes C (2011). Activity space environment and dietary and physical activity behaviours: a pilot study. Health Place.

[72] Burgoine Thomas, Monsivais Pablo (2013). Characterising food environment exposure at home, at work, and along commuting journeys using data on adults in the UK. International Journal of Behavioral Nutrition and Physical Activity.

[73] Hutcheon J. A., Chiolero A., Hanley J. A. (2010). Random measurement error and regression dilution bias. BMJ.

[74] Kirkpatrick SI, Reedy J, Butler EN, Dodd KW, Subar AF, Thompson FE, McKinnon RA (2014). Dietary assessment in food environment research: a systematic review. Am J Prev Med.

[75] Adler NE, Ostrove JM (1999). Socioeconomic status and health: what we know and what we don't. Ann N Y Acad Sci.

[76] Bradford Hill A (1965). The environment and disease: association or causation?. Proc Royal Soc Med.

