# Correction to: Examining the interaction of fast-food outlet exposure and income on diet and obesity: evidence from 51,361 UK biobank participants

**DOI:** 10.1186/s12966-018-0713-1

**Published:** 2018-09-25

**Authors:** Thomas Burgoine, Chinmoy Sarkar, Chris J. Webster, Pablo Monsivais

**Affiliations:** 1 UKCRC Centre for Diet and Activity Research (CEDAR), MRC Epidemiology Unit, University of Cambridge School of Clinical Medicine, Box 285 Institute of Metabolic Science, Cambridge Biomedical Campus, Cambridge, CB2 0QQ UK; 2 Healthy High Density Cities Lab, HKUrbanLab, University of Hong Kong, Knowles Building, Pokfulam Road, Hong Kong, Special Administrative Region, China; 3 Present Address: Department of Nutrition and Exercise Physiology, Elson S. Floyd College of Medicine, Washington State University, Spokane, Washington, USA

## Correction

The original article [1] contained a number of minor copyediting errors in the main body. These errors have now been corrected.

Furthermore, these errors were mistakenly introduced by the Production team managing this article and, as such were not the fault of the authors.
