# Supplementary material for: Examining the interaction of fast-food outlet exposure and income on diet and obesity: evidence from 51,361 UK Biobank participants
Source: Int J Behav Nutr Phys Act. 2018 Jul 24;15:71. doi: 10.1186/s12966-018-0699-8 (PMC6497220; doi:10.1186/s12966-018-0699-8)
Supplement: Supplementary file 1 — Flow diagram for UK Biobank sample restriction, for body weight-, processed meat consumption- and percentage body fat-based analyses reported in this study. (DOCX 37 kb) [file 12966_2018_699_MOESM1_ESM.docx]

**Additional File 1:** Flow diagram for UK Biobank sample restriction, for body weight-, processed meat consumption- and percentage body fat-based analyses reported in this study.

**UK Biobank Cohort**

Participants attending UK Biobank assessment centres in Greater London (Barts, Hounslow and Croydon): n=68 850

Missing data on neighbourhood food outlet exposure: n=16 494

Missing data across other covariates (not processed meat consumption, percentage body fat): n=995

**Analytic sample** for body mass index models: n=51 361

Missing data on processed meat consumption: n=271

**Analytic sample** for processed meat consumption models: n=51 090

Missing data on percentage body fat: n=595

**Analytic sample** for percentage body fat models: n=50 766
