# Supplementary material for: Examining the interaction of fast-food outlet exposure and income on diet and obesity: evidence from 51,361 UK Biobank participants
Source: Int J Behav Nutr Phys Act. 2018 Jul 24;15:71. doi: 10.1186/s12966-018-0699-8 (PMC6497220; doi:10.1186/s12966-018-0699-8)
Supplement: Supplementary file 2 — UK Biobank participants attending Greater London assessment centres (Barts, Croydon, Hounslow; n = 68,850) and UK Biobank analytic sample (n = 51,361) demographic comparisons. (DOCX 20 kb) [file 12966_2018_699_MOESM2_ESM.docx]

**Additional File 2:** UK Biobank participants attending Greater London assessment centres (Barts, Croydon, Hounslow; n=68 850) and UK Biobank analytic sample (n=51 361) demographic comparisons.

|  | **Greater London UK Biobank full sample (n=68 850)** | **Greater London UK Biobank analytic sample (n=51 361)** |
| --- | --- | --- |
|  | Mean (SD) or % | Mean (SD) or % |
| Mean Age (SD) | 56.1 (8.2) | 56.0 (8.2) |
| Sex, men (%) | 44.4 | 43.9 |
| Ethnicity, white (%) | 81.1 | 79.7 |
| Mean BMI, kg/m^2^ (SD) | 27.0 (4.9) | 26.9 (4.9) |
| Weight status, obese ^a^ (%) | 22.0 | 21.8 |
| Mean Percentage Body Fat ^b^ (SD) | 30.8 (8.6) | 30.8 (8.6) |
| Processed meat consumption ^c^, >1/wk (%) | 28.4 | 27.9 |
| Smoking Status, current or ex (%) | 46.4 | 46.3 |
| Highest Education, Higher ^d^ (%) | 57.0 | 58.5 |
| Household Income, >£100 000 (%) | 9.9 | 10.5 |
| ^a^ Body mass index ≥30 kg/m^2^ \| ^b^ Measured using bioelectrical impedance analysis \| ^c^ Includes bacon, ham, sausages, meat pies, kebabs, burgers, chicken nuggets \| ^d^ Participants completing >13 years of education. | | |
