# Supplementary material for: Examining the interaction of fast-food outlet exposure and income on diet and obesity: evidence from 51,361 UK Biobank participants
Source: Int J Behav Nutr Phys Act. 2018 Jul 24;15:71. doi: 10.1186/s12966-018-0699-8 (PMC6497220; doi:10.1186/s12966-018-0699-8)
Supplement: Supplementary file 3 — Characteristics of participants in the UK Biobank sample, UK (n = 51,361), overall and stratified by household income. (DOCX 24 kb) [file 12966_2018_699_MOESM3_ESM.docx]

| **Additional File 3:** Characteristics of participants in the UK Biobank sample, UK (n=51 361), overall and stratified by household income. | | | | | | |
| --- | --- | --- | --- | --- | --- | --- |
|  | **Household income, £/year** | | | | |  |
|  | <31,000 | 31,000 – 51,999 | 52,000 – 100,000 | >100,000 | Don’t know or  Prefer not to say | **All** |
| Age, years | 57.8 (8.2) | 55.8 (8.1) | 54.3 (7.8) | 52.6 (7.6) | 57.0 (8.3) | 56.0 (8.2) |
| Men (no. (%)) | 6967 (42.0) | 4686 (43.8) | 5390 (48.7) | 2726 (50.7) | 2756 (36.2) | 22 525 (43.9) |
| Ethnicity (no. (%)) |  |  |  |  |  |  |
| White | 12 246 (73.8) | 8851 (82.8) | 9835 (88.9) | 5018 (93.3) | 4982 (65.4) | 40 932 (79.7) |
| Asian or Asian British | 1543 (9.3) | 618 (5.8) | 492 (4.4) | 135 (2.5) | 1046 (13.7) | 3834 (7.5) |
| Black or Black British | 1544 (9.3) | 669 (6.3) | 355 (3.2) | 45 (0.8) | 746 (9.8) | 3359 (6.5) |
| Other | 742 (4.5) | 268 (2.5) | 167 (1.5) | 66 (1.2) | 441 (5.8) | 1684 (3.3) |
| Don’t know or Prefer not to say | 116 (0.7) | 54 (0.5) | 34 (0.3) | 13 (0.2) | 189 (2.5) | 406 (0.8) |
| Highest education (no. (%)) |  |  |  |  |  |  |
| Compulsory (≤11 y of education) or Other ^a^ | 6116 (36.9) | 2156 (20.2) | 1313 (11.9) | 244 (0.0) | 2909 (38.2) | 12 738 (24.8) |
| Further (12-13 y of education) | 2895 (17.4) | 1851 (17.3) | 1476 (13.3) | 395 (7.3) | 1105 (14.5) | 7722 (15.0) |
| Higher (>13 y of education) | 7381 (44.5) | 6650 (62.2) | 8267 (74.7) | 4739 (88.1) | 3020 (39.6) | 30 057 (58.5) |
| Prefer not to say | 207 (1.3) | 33 (0.3) | 13 (0.1) | 2 (0.0) | 589 (7.7) | 844 (1.6) |
| Current or ex-smoker (no. (%)) | 8058 (48.6) | 5011 (46.9) | 5185 (46.8) | 2429 (45.2) | 3076 (40.4) | 23 759 (46.3) |
| Anthropometric and Diet Outcomes |  |  |  |  |  |  |
| BMI, kg/m^2^ | 27.4 (5.2) | 26.9 (4.8) | 26.5 (4.5) | 25.8 (4.1) | 27.4 (5.1) | 26.9 (4.9) |
| Body Fat, % | 31.9 (8.8) | 30.8 (8.4) | 29.5 (8.2) | 28.0 (7.8) | 32.5 (8.6) | 30.8 (8.6) |
| Obese, BMI≥30 (no. (%)) | 4316 (26.0) | 2213 (20.7) | 1983 (17.9) | 717 (13.3) | 1974 (25.9) | 11 203 (21.8) |
| Frequent processed meat consumption ^e^ | 4578 (27.6) | 2975 (27.8) | 3220 (29.1) | 1484 (27.6) | 1978 (26.0) | 14 235 (27.7) |
| **Food Environment Exposures** ^b^ |  |  |  |  |  |  |
| Fast-food outlets | 39.8 (26.8) | 38.0 (26.4) | 39.2 (27.7) | 42.7 (29.8) | 37.1 (25.8) | 39.2 (27.1) |
| Other food outlets ^c^ | 220.2 (269.0) | 208.6 (241.5) | 232.0 (258.1) | 317.7 (319.9) | 205.0 (240.9) | 228.3 (265.0) |
| Fast-food outlet proportion, % ^d^ | 19.5 (7.9) | 18.9 (7.8) | 17.7 (7.7) | 14.4 (6.9) | 19.3 (7.9) | 18.4 (7.9) |
| Data are mean (standard deviation) unless otherwise stated; percentages represent column percentage \| ^a^ Those reporting ‘Other’ education will include those with no and non-British qualifications \| ^b^ Counts of food outlets within 1 mile Euclidean (straight line) radius buffers of home address \| ^c^ Sum of counts of Supermarkets, Restaurants, Convenience stores, Cafes and Specialist stores \| ^d^ Fast-food outlets expressed as a proportion of the sum of counts of Fast-food outlets, Supermarkets, Restaurants, Convenience Stores, Cafes and Specialist stores \| ^e^ Frequent consumption was defined as more than once per week; processed meat includes bacon, ham, sausages, meat pies, kebabs, burgers, chicken nuggets. | | | | | | |
