# Supplementary material for: Examining the interaction of fast-food outlet exposure and income on diet and obesity: evidence from 51,361 UK Biobank participants
Source: Int J Behav Nutr Phys Act. 2018 Jul 24;15:71. doi: 10.1186/s12966-018-0699-8 (PMC6497220; doi:10.1186/s12966-018-0699-8)
Supplement: Supplementary file 4 — Associations of quartiles of fast-food outlet proportion with body fat percentage (estimated using a multivariable linear regression model, n = 50,766) in the Greater London UK Biobank sample. (DOCX 20 kb) [file 12966_2018_699_MOESM4_ESM.docx]

| **Additional File 4:** Associations of quartiles of fast-food outlet proportion with body fat percentage (estimated using a multivariable linear regression model, n=50 766) in the Greater London UK Biobank sample. | | | | | | | | | |
| --- | --- | --- | --- | --- | --- | --- | --- | --- | --- |
|  |  | **Model 1** ^a^ |  | **Model 2** ^b^ |  | **Model 3** ^c^ |  | **Model 4** ^d^ |  |
|  | **Quartile** ^e^ | β | 95% CI | β | 95% CI | β | 95% CI | β | 95% CI |
| **Difference in Body Fat ^f^ (%)** | Q1 (0.0-12.7%) | ref |  | ref |  | ref |  | ref |  |
|  | Q2 (12.7-16.9%) | 0.56** | 0.35, 0.77 | 0.55** | 0.39, 0.70 | 0.39** | 0.23, 0.54 | 0.35** | 0.19, 0.51 |
|  | Q3 (16.9-23.7%) | 1.51** | 1.30, 1.72 | 1.41** | 1.25, 1.57 | 1.07** | 0.91, 1.24 | 1.01** | 0.83, 1.18 |
|  | Q4 (23.7-44.6%) | 2.17** | 1.96, 2.38 | 1.89** | 1.74, 2.05 | 1.45** | 1.26, 1.63 | 1.37** | 1.17, 1.56 |
| ** *p*<0.001 \|  ^a^ Model 1 is an unadjusted model \|  ^b^ Model 2 adjusts for age, sex, ethnicity, smoking status \|  ^c^ Model 3 additionally adjusts for household income, number in household, highest educational attainment and UK Biobank assessment centre attended \|  ^d^ Model 4 additionally adjusts for sum of counts of Supermarkets, Restaurants, Convenience stores, Cafes and Specialist Stores within 1 mile Euclidean (straight line) radius buffers of home address \|  ^e^ Q1=quartile with lowest fast-food outlet proportion in home neighbourhood (% range) – Q4=quartile with greatest fast-food outlet proportion in home neighbourhood (min max %) \|  ^f^ Measured using bioelectrical impedance analysis. | | | | | | | | | |
