# Supplementary material for: Examining the interaction of fast-food outlet exposure and income on diet and obesity: evidence from 51,361 UK Biobank participants
Source: Int J Behav Nutr Phys Act. 2018 Jul 24;15:71. doi: 10.1186/s12966-018-0699-8 (PMC6497220; doi:10.1186/s12966-018-0699-8)
Supplement: Supplementary file 5 — Adjusted risk ratios (RRs) describing the associations of quartiles of fast-food outlet proportion with body mass index (estimated using a multivariable linear regression model, n = 51,361), obesity (estimated using a binomial logistic regression model n = 51,361), and frequent consumption of processed meat (estimated using a binomial logistic regression model, n = 51,090) in the Greater London UK Biobank sample. (DOCX 22 kb) [file 12966_2018_699_MOESM5_ESM.docx]

| **Additional File 5:** Adjusted risk ratios describing the associations of quartiles of fast-food outlet proportion with body mass index (estimated using a multivariable linear regression model, n=51 361), obesity (estimated using a binomial logistic regression model n=51 361), and frequent consumption of processed meat (estimated using a binomial logistic regression model, n=51 090) in the Greater London UK Biobank sample. | | | | | | | | | |
| --- | --- | --- | --- | --- | --- | --- | --- | --- | --- |
|  |  | **Model 1** ^a^ |  | **Model 2** ^b^ |  | **Model 3** ^c^ |  | **Model 4** ^d^ |  |
|  | **Quartile** ^e^ | RR | 95% CI | RR | 95% CI | RR | 95% CI | RR | 95% CI |
| **Odds of obesity (BMI≥30 kg/m^2^)** | Q1 (0.0-12.7%) | ref |  | ref |  | ref |  | ref |  |
|  | Q2 (12.7-16.9%) | 1.22** | 1.16, 1.29 | 1.20** | 1.14, 1.26 | 1.15** | 1.09, 1.21 | 1.15** | 1.09, 1.21 |
|  | Q3 (16.9-23.7%) | 1.49** | 1.42, 1.57 | 1.42** | 1.36, 1.50 | 1.32** | 1.25, 1.39 | 1.31** | 1.24, 1.38 |
|  | Q4 (23.7-44.6%) | 1.62** | 1.54, 1.70 | 1.51** | 1.44, 1.59 | 1.39** | 1.31, 1.47 | 1.37** | 1.29, 1.46 |
|  |  |  |  |  |  |  |  |  |  |
|  |  | **Model 1** ^a^ |  | **Model 2** ^f^ |  | **Model 3** ^c^ |  | **Model 4** ^d^ |  |
|  | **Quartile** ^e^ | RR | 95% CI | RR | 95% CI | RR | 95% CI | RR | 95% CI |
| **Odds of frequent consumption of processed meat** ^g^ **(> once per week)** | Q1 (0.0-12.7%) | ref |  | ref |  | ref |  | ref |  |
|  | Q2 (12.7-16.9%) | 1.07* | 1.03, 1.12 | 1.10** | 1.06, 1.15 | 1.07* | 1.03, 1.11 | 1.07* | 1.02, 1.11 |
|  | Q3 (16.9-23.7%) | 1.13** | 1.09, 1.18 | 1.19** | 1.14, 1.24 | 1.13** | 1.08, 1.18 | 1.12** | 1.07, 1.17 |
|  | Q4 (23.7-44.6%) | 1.20** | 1.16, 1.25 | 1.28** | 1.23, 1.33 | 1.19** | 1.14, 1.25 | 1.18** | 1.13, 1.24 |
| Adjusted RRs calculated using Stata post-estimation command adjrr \| **p*<0.05; ** *p*<0.001 \| ^a^ Model 1 is an unadjusted model \| ^b^ Model 2 adjusts for age, sex, ethnicity, smoking status \| ^c^ Model 3 additionally adjusts for household income, number in household, highest educational attainment and UK Biobank assessment centre attended \| ^d^ Model 4 additionally adjusts for sum of counts of Supermarkets, Restaurants, Convenience stores, Cafes and Specialist Stores within 1 mile Euclidean (straight line) radius buffers of home address \| ^e^ Q1=quartile with lowest fast-food outlet proportion in home neighbourhood (min-max %) – Q4=quartile with greatest fast-food outlet proportion in home neighbourhood (min-max %) \| ^f^ Model 2 adjusts for age, sex, ethnicity \| ^g^ Includes bacon, ham, sausages, meat pies, kebabs, burgers, chicken nuggets. | | | | | | | | | |
