# Supplementary material for: Examining the interaction of fast-food outlet exposure and income on diet and obesity: evidence from 51,361 UK Biobank participants
Source: Int J Behav Nutr Phys Act. 2018 Jul 24;15:71. doi: 10.1186/s12966-018-0699-8 (PMC6497220; doi:10.1186/s12966-018-0699-8)
Supplement: Supplementary file 6 — Associations of household income with body fat percentage (estimated using a multivariable linear regression model, n = 50,766) in the Greater London UK Biobank sample. (DOCX 20 kb) [file 12966_2018_699_MOESM6_ESM.docx]

| **Additional File 6:** Associations of household income with body fat percentage (estimated using a multivariable linear regression model, n=50 766) in the Greater London UK Biobank sample. | | | | | | | | | |
| --- | --- | --- | --- | --- | --- | --- | --- | --- | --- |
|  | **Household Income**  **(£/year)** | **Model 1** ^a^ |  | **Model 2** ^b^ |  | **Model 3** ^c^ |  | **Model 4** ^d^ |  |
|  |  | β | 95% CI | β | 95% CI | β | 95% CI | β | 95% CI |
| **Difference in Body Fat** ^e^ **(%)** | > 100 000 | ref |  | ref |  | ref |  | ref |  |
|  | 52 000-100 000 | 1.55** | 1.18, 1.74 | 0.93** | 0.72, 1.14 | 0.69** | 0.48, 0.90 | 0.53** | 0.32, 0.74 |
|  | 31 000-51 999 | 2.73** | 2.45, 3.01 | 1.39** | 1.18, 1.60 | 0.96** | 0.74, 1.17 | 0.74** | 0.52, 0.95 |
|  | < 31 000 | 3.78** | 3.52, 4.05 | 1.78** | 1.57, 1.99 | 1.06** | 0.85, 1.28 | 0.83** | 0.61, 1.04 |
| ** *p*<0.001 \|  ^a^ Model 1 adjusts for number in household \|  ^b^ Model 2 additionally adjusts for age, sex, ethnicity, smoking status \|  ^c^ Model 3 additionally adjusts for highest educational attainment and UK Biobank assessment centre attended \|  ^d^ Model 4 additionally adjusts for fast-food outlet proportion and the sum of counts of Supermarkets, Restaurants, Convenience stores, Cafes and Specialist Stores within 1 mile Euclidean (straight line) radius buffers of home address \|  ^e^ Measured using bioelectrical impedance analysis. | | | | | | | | | |
