# Supplementary material for: Examining the interaction of fast-food outlet exposure and income on diet and obesity: evidence from 51,361 UK Biobank participants
Source: Int J Behav Nutr Phys Act. 2018 Jul 24;15:71. doi: 10.1186/s12966-018-0699-8 (PMC6497220; doi:10.1186/s12966-018-0699-8)
Supplement: Supplementary file 7 — Adjusted risk ratios (RRs) describing the associations of household income with body mass index (estimated using a multivariable linear regression model, n = 51,361), obesity (estimated using a binomial logistic regression model, n = 51,361), and frequent consumption of processed meat (estimated using a binomial logistic regression model, n = 51,090) in the Greater London UK Biobank sample. (DOCX 22 kb) [file 12966_2018_699_MOESM7_ESM.docx]

| **Additional File 7:** Adjusted risk ratios (RRs) describing the associations of household income with body mass index (estimated using a multivariable linear regression model, n=51 361), obesity (estimated using a binomial logistic regression model, n=51 361), and frequent consumption of processed meat (estimated using a binomial logistic regression model, n=51 090) in the Greater London UK Biobank sample. | | | | | | | | | |
| --- | --- | --- | --- | --- | --- | --- | --- | --- | --- |
|  | **Household Income**  **(£/year)** | **Model 1** ^a^ |  | **Model 2** ^b^ |  | **Model 3** ^c^ |  | **Model 4** ^d^ |  |
|  |  | RR | 95% CI | RR | 95% CI | RR | 95% CI | RR | 95% CI |
| **Odds of obesity (BMI≥30 kg/m^2^)** | > 100 000 | ref |  | ref |  | ref |  | ref |  |
|  | 52 000-100 000 | 1.34** | 1.24, 1.45 | 1.29** | 1.20, 1.40 | 1.22** | 1.13, 1.32 | 1.17** | 1.09, 1.27 |
|  | 31 000-51 999 | 1.55** | 1.44, 1.68 | 1.44** | 1.34, 1.56 | 1.31** | 1.22, 1.42 | 1.24** | 1.15, 1.34 |
|  | < 31 000 | 1.95** | 1.81, 2.10 | 1.75** | 1.62, 1.88 | 1.49** | 1.38, 1.60 | 1.40** | 1.30, 1.51 |
|  |  |  |  |  |  |  |  |  |  |
|  | **Household Income**  **(£/year)** | **Model 1** ^a^ |  | **Model 2** ^e^ |  | **Model 3** ^c^ |  | **Model 4** ^d^ |  |
|  |  | RR | 95% CI | RR | 95% CI | RR | 95% CI | RR | 95% CI |
| **Odds of frequent consumption of processed meat** ^f^ **(> once per week)** | > 100 000 | ref |  | ref |  | ref |  | ref |  |
|  | 52 000-100 000 | 1.06* | 1.00, 1.11 | 1.12** | 1.06, 1.18 | 1.09* | 1.03, 1.15 | 1.07* | 1.01, 1.13 |
|  | 31 000-51 999 | 1.01 | 0.96, 1.07 | 1.16** | 1.10, 1.23 | 1.13** | 1.07, 1.19 | 1.10* | 1.04, 1.56 |
|  | < 31 000 | 1.01 | 0.96, 1.06 | 1.25** | 1.18, 1.31 | 1.20** | 1.33, 1.26 | 1.16** | 1.10, 1.23 |
| Adjusted RRs calculated using Stata post-estimation command adjrr \| **p*<0.05; ** *p*<0.001 \|  ^a^ Model 1 adjusts for number in household \|  ^b^ Model 2 additionally adjusts for age, sex, ethnicity, smoking status \|  ^c^ Model 3 additionally adjusts for highest educational attainment and UK Biobank assessment centre attended \|  ^d^ Model 4 additionally adjusts for fast-food outlet proportion and the sum of counts of Supermarkets, Restaurants, Convenience stores, Cafes and Specialist Stores within 1 mile Euclidean (straight line) radius buffers of home address \|  ^e^ Model 2 adjusts for age, sex, ethnicity \|  ^f^ Includes bacon, ham, sausages, meat pies, kebabs, burgers, chicken nuggets. | | | | | | | | | |
